# High-affinity ssDNA aptamer and chemiluminescent aptasensor for TIMP-1 detection in human serum

**DOI:** 10.1007/s44211-024-00673-w

**Published:** 2025-01-01

**Authors:** Qin Wang, Yanli Li, Lige Yao, Huiqin Li, Liuyan Zhang, Yingjie Wang, Jiayin Li, Tian Chen, Kun Chai, Junli Gao, Junshun Gao, Li Su, Xueming Li

**Affiliations:** 1 The Third People’s Hospital of Zhengzhou, Zhengzhou, 450000 China; 2 The First People’s Hospital of Shangqiu, Shangqiu, 476000 China; 3 Hangzhou Cosmos Wisdom Mass Spectrometry Center of Zhejiang University Medical School, Hangzhou, 311200 China

**Keywords:** TIMP-1, Aptamer selection, Chemiluminiscence, Aptasensor, Biomarker, Cancer

## Abstract

TIMP-1 (Tissue Inhibitor of Metalloproteinases-1) is a protein involved in regulating extracellular matrix (ECM) degradation. It is recognized as a significant biomarker for cancer diagnosis. This study aimed to develop and characterize a single-stranded DNA (ssDNA) aptamer targeting human TIMP-1 protein with high affinity and specificity. A magnetic beads-based SELEX process combined with qPCR was used to select aptamers over seven rounds. The enriched ssDNA library was analyzed using high-throughput sequencing to identify candidate sequences, and these sequences were characterized using surface plasmon resonance (SPR) and binding assays to evaluate their affinity and specificity. The selected ssDNA aptamer demonstrated a dissociation equilibrium constant (*K*_D_) of 0.41 nM and a very slow off-rate, enabling effective capture of TIMP-1 in serum samples. Furthermore, a chemiluminescent aptasensor was developed for TIMP-1 detection, which exhibited high specificity and a broad linear detection range from 1 to 500 ng/mL in human serum. The developed ssDNA aptamer targeting TIMP-1 shows high affinity and specificity, and the chemiluminescent aptasensor demonstrates promising potential for clinical diagnosis of TIMP-1 levels in human serum.

**Graphical abstract:**

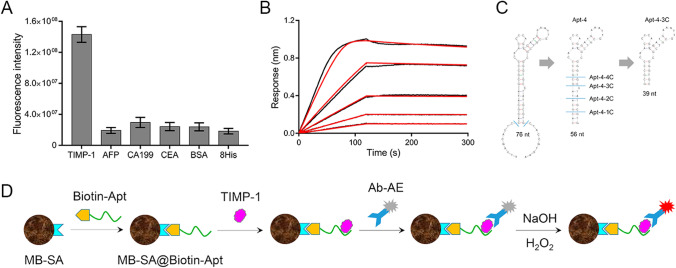

## Introduction

Tissue inhibitor of metalloproteinases 1 (TIMP-1), a member of the TIMP family, is a glycoprotein as a natural tissue inhibitor of matrix metalloproteinases (MMPs) [1, 2]. MMPs are a group of peptidases involved in the degradation of the ECM and play a vital role in the normal physiology of connective tissue during development, morphogenesis, and wound healing. Due to its inhibitory role against the MMPs, the TIMP-1 protein can promote cell proliferation and may also have an anti-apoptotic function [3]. Numerous studies have shown that TIMP-1 is associated with cancer-cell invasion and metastasis, such as pancreatic, gastric, colorectal, and breast cancer [4–8]. Therefore, detecting TIMP-1 as a vital biomarker is of great significance.

Currently, the routine analysis methods used for TIMP-1 are mainly antibody-based immunoassays [9–11]. Aptamers are single-stranded oligonucleotides (DNA/RNA) that can fold to form specific tertiary structures with high binding affinity and specificity for their targets. Compared with antibodies, aptamers can be screened through in vitro selection procedures termed systematic evolution of ligands by exponential enrichment (SELEX) and produced through chemical synthesis without using animals or cells. Aptamers can be easily chemically modified and conveniently operated with no batch difference. These excellent characteristics make aptamers gain increasing attention in different fields, such as environmental monitoring, food security, disease diagnosis, and therapies [12–16]. Up to now, the aptamer targeting for TIMP-1 has not yet been reported in the literature. In this research, the single stranded DNA (ssDNA) aptamer targeting for TIMP-1 was discovered, showing high specificity, affinity, and a very slow off-rate. Furthermore, a chemiluminescent aptasensor was developed to detect TIMP-1 concentration in human serum.

## Material and methods

### Material and reagents

The aptamer selection reagents, such as the ssDNA libraries, PCR primers, PCR mix, 1-ethyl-3-(3-dimethylaminopropyl) carbodiimide hydrochloride (EDC), N-hydroxysuccinimide (NHS), and carboxylated magnetic beads were purchased from Anhui Aptamy Biotechnology Co., Ltd. (Hefei, China). His tagged TIMP-1 protein and 8His peptide were purchased from GenScript (Nanjing, China). The aptamers were synthesized by Genewiz (Suzhou, China). Dulbecco’s phosphate buffered saline solution (DPBS) contained 138 mM NaCl, 2.67 mM KCl, 8.1 mM Na_2_HPO_4_, 1.47 mM KH_2_PO_4_, 1 mM CaCl_2_, and 0.5 mM MgCl_2_ was used as the aptamer selection buffer. All sequences are listed in Table 1. Human negative serum was obtained from Santa Scott Biotechnology Co.,ltd (Nanjing, China).

**Table 1:** The sequences used in this paper

DNA species	Sequences (5′ → 3′)
DNA library	TTC AGC ACT CCA CGC ATA GC-36N-CCT ATG CGT GCT ACC GTG AA
Forward primer	TTC AGC ACT CCA CGC ATA GC
Forward primer-FAM	FAM-TTC AGC ACT CCA CGC ATA GC
Reverse primer	TTC ACG GTA GCA CGC ATA GG
Reverse primer-polyA	A20-Spacer 18-TTC ACG GTA GCA CGC ATA GG
Apt-4	CAC GCA TAG CGC CCC AAC CCC AGT CGA TGA AAT TGC GTC GCG GGT GCC TAT GCG TG
Apt-4-1C	GCA TAG CGC CCC AAC CCC AGT CGA TGA AAT TGC GTC GCG GGT GCC TAT GC
Apt-4-2C	TAG CGC CCC AAC CCC AGT CGA TGA AAT TGC GTC GCG GGT GCC TA
Apt-4-3C	G CGC CCC AAC CCC AGT CGA TGA AAT TGC GTC GCG GGT GC
Apt-4-4C	GC CCC AAC CCC AGT CGA TGA AAT TGC GTC GCG GGT

### Aptamer selection

An ssDNA oligonucleotide library with 36 random nucleotide sequences was used to screen ssDNA aptamers targeting the TIMP-1 protein. The SELEX procedure was performed as the literature report with some modifications [17]. Briefly, the TIMP-1 protein was first immobilized onto the carboxylated magnetic beads through the amine coupling procedure. Then the beads were incubated with the ssDNA library consisting of about 10^15^ sequences in DPBS buffer. The unbound sequences in the supernate were removed through magnetic separation, and the bound sequences on the beads were collected and eluted. The recovered sequences were amplified by emulsion PCR (ePCR) to decrease the amplification preferences under the following conditions [18]: denaturation at 95 ℃ for 1 min, annealing at 60 ℃ for 1 min, and elongation at 72 ℃ for 1 min. After the ePCR amplification, n-butanol was added to break up the emulsion. The collected dsDNA was converted into ssDNA via denaturing urea polyacrylamide gel electrophoresis and dialyzed in DPBS buffer for the next selection round. During each selection round, a quantitative PCR (qPCR) strategy was performed to monitor the progress of aptamer selection [17]. Before each selection round, a counter-selection was performed using 8His peptide-modified magnetic beads to avoid screening the aptamer targeting the His-tag of TIMP-1 protein. After seven rounds of selection, the enriched ssDNA pool was identified by high-throughput sequencing.

### qPCR

qPCR experiments were performed on Bio-Rad CFX96 Real-Time PCR System according to the manufacturer’s instructions. All qPCR reactions were performed in 30 μL reaction volumes containing 0.5 μM primers, 0.2 μM dNTP, 20 U/mL Pfu DNA polymerase, 4% EvaGreen, and 2 μL DNA template under the following thermal cycling conditions: denaturation at 95 ℃ for 30 s, annealing at 60 ℃ for 30 s, and elongation at 72 ℃ for 30 s. The qPCR data were analyzed using the Bio-Rad CFX Maestro software that came with the instrument.

### Structure analysis

All DNA secondary structures of the selected aptamers were analyzed using the mfold web server [19] (http://www.unafold.org/mfold/applications/dna-folding-form.php).

### Surface plasmon resonance analysis

The binding screening of the aptamer candidates was performed using the Biacore T200 biosensor system. DPBS buffer was used as the running buffer, and the flow rate was set at 20 µL/min. A CM5 sensor chip was used, where TIMP-1 protein was immobilized through the EDC/NHS-based amine coupling procedure. Each aptamer candidate in DPBS at 1 μM was introduced onto the sensor surface for binding measurement monitoring the association and dissociation for 2 min, respectively. After each measurement, the sensor surface was regenerated using 1 M NaCl. A reference channel on which 8His peptide was immobilized was used for background subtraction.

### Nitrocellulose filter binding assay

The FAM-labeled aptamers were incubated with proteins in a final volume of 200 μL for 1 h. After the incubation, the mixtures were filtered through a nitrocellulose membrane in a Bio-Dot SF microfiltration apparatus (Bio-Rad) and rinsed with DPBS buffer. Then the nitrocellulose membrane was scanned through the ChemiDoc MP imaging system (Bio-Rad), and the binding aptamers were quantified using the Image Lab software (V 6.0.1, Bio-Rad).

### Bio-layer interferometry assay (BLI)

The affinity and kinetics properties of the interaction between aptamer and TIMP-1 were acquired using an Octet R4 system and analyzed using the Octet Analysis Studio software version 12.2. The biotinylated aptamer at 500 nM was loaded onto a streptavidin-coated biosensor until a 0.8 nm response was reached. Serial dilutions of TIMP-1 in binding buffer (DPBS with 0.025% Tween 20) were prepared for the interaction measurement (association 120 s and dissociation 180 s). The sensorgrams were analyzed by subtracting the reference well and global fitting using a 1:1 binding mode with mass transport.

### Chemiluminescent aptasensor immunoassay

In chemiluminescent aptasensor immunoassay, 500 pmol biotinylated aptamers (Biotin-Ap) were incubated with 1 mg streptavidin magnetic beads (MB-SA) for 1 h with slow rotation at room temperature. After incubation, the magnetic beads (MB-SA@Biotin-Apt) were washed with DPBS 3 times and ready for chemiluminescent aptasensor immunoassay. The acridinium ester labeled TIMP-1 antibody (Ab-AE) was prepared according to the literature [20]. The MB-SA@Biotin-Apt, Ab-AE, and TIMP-1 samples were loaded into an automated chemiluminescent immunoassay system (Keysmile SMART 6500S, Chongqing, China) and tested according to the manual.

### Enzyme-linked immunosorbent assay (ELISA)

For comparison, the TIMP-1 concentrations in human sera samples were also tested using a Human TIMP-1 Quantikine ELISA Kit (R&D Systems) in which the plate was pre-coated with human TIMP-1 monoclonal antibody. The assay was performed according to the manufacturer’s instructions. Briefly, 50 μL samples (80-fold dilution) or standards were added to the plate in duplicate and incubated for 2 h. After a brief wash, 200 μL human TIMP-1 polyclonal antibody conjugated to horseradish peroxidase was applied to each well for 1 h. The substrate solution was subsequently incubated for 30 min and terminated with 2N H_2_SO_4_. The optical density was determined using a microplate reader at 450 nm.

For the inhibition experiment, TIMP-1 samples were pre-incubated with threefold mol of the TIMP-1 antibody (TIMP-1 + Ab) or aptamer (TIMP-1 + Apt) for 1 h. Then the TIMP-1 sample, TIMP-1 + Ab mixture, and TIMP-1 + Apt mixture were transferred to the plate. The TIMP-1 antibody and aptamer as well as a blank buffer without TIMP-1 were used as negative control. After incubation for 2 h, the plate was washed and a human TIMP-1 polyclonal antibody conjugated to horseradish peroxidase was added. Other steps were the same as above.

## Results

### Aptamer selection and characterization

**Fig. 1:**
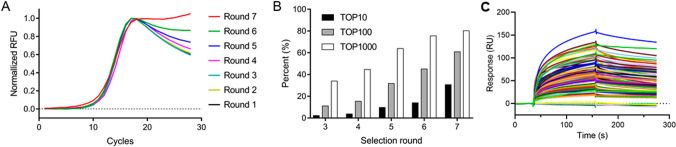
**A** Amplification curve of every round of ssDNA library during aptamer selection. **B** The abundance of top 10, 100, and 1000 sequences in the ssDNA library. **C** Binding characterization of the 96 candidate sequences by SPR

The aptamer selection process followed a magnetic beads-based SELEX approach combined with qPCR for monitoring selection progress. Considering the TIMP-1 protein’s positive charge at physiological pH (pI 8.0) [21] and the potential non-specific electrostatic interactions with negatively charged ssDNA, a high ionic strength buffer containing 500 mM sodium chloride was employed. Additionally, 50% serum was incorporated into the selection buffer to enhance specificity, stability, and adaptability of the aptamers. Figure 1A illustrates the qPCR amplification curves of the ssDNA library in each selection round. The fluorescence drop after the 18th PCR cycle gradually disappears along with the selection round, indicating reduced ssDNA library diversity [17]. High-throughput sequencing confirmed the enrichment, with top sequences displaying increased abundance throughout the selection rounds (Fig. 1B). After seven selection rounds, the enriched ssDNA library was sequenced, yielding 96 abundant sequences for chemical synthesis and SPR binding characterization. Figure 1C demonstrates the varying binding properties of these sequences to TIMP-1, with Apt-4 showing the highest binding signal.

### Specificity and binding kinetics of Apt-4

**Fig. 2:**
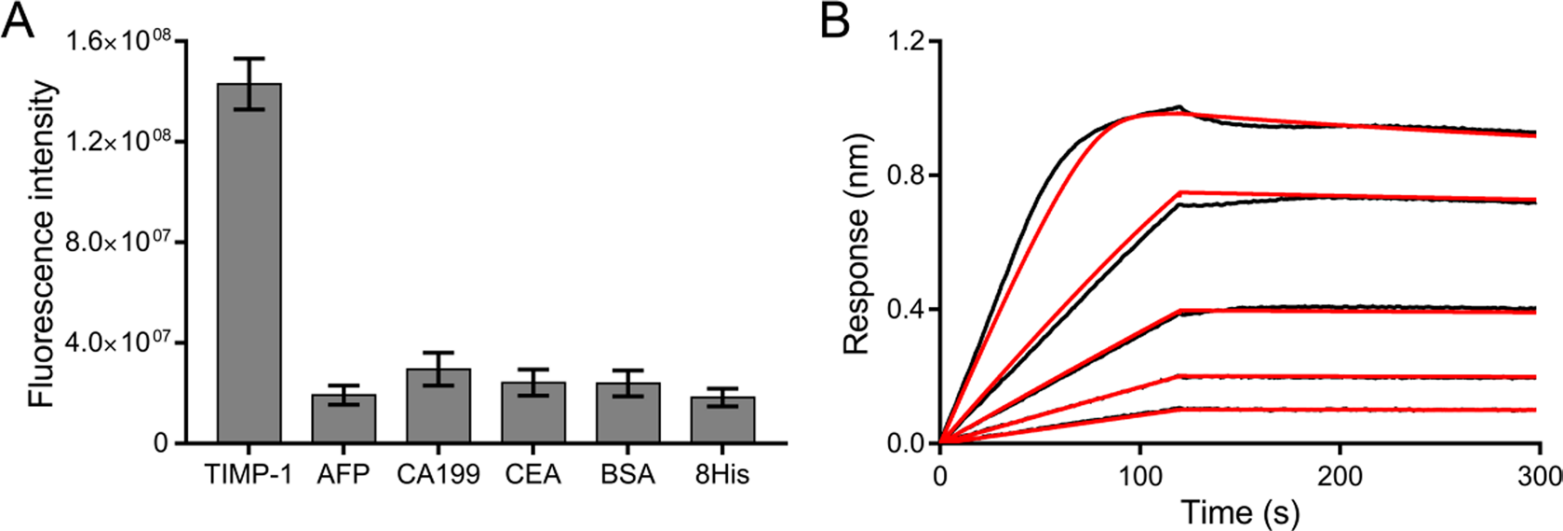
The specificity and kinetics of the aptamer. **A** The specificity evaluation was performed using a nitrocellulose filter binding assay. AFP is alpha-fetoprotein. CA199 is carbohydrate antigen 19–9. CEA is carcinoembryonic antigen. BSA is bovine serum albumin. **B** Biolayer interferometry (BLI) sensorgrams for aptamer-TIMP-1 interaction. TIMP-1 was tested at concentrations of 6.65, 13.3, 26.6, 53.2, and 106.4 nM. The responses were recorded (black curves) and were globally fitted to a theoretical 1:1 binding model with a mass transport parameter (red curves), yielding the association rate constant (*k*_on_ = 1.30 × 10^6^ M^−1^ s^−1^), dissociation rate constant (*k*_off_ = 5.36 × 10^−4^ s^−1^), and dissociation equilibrium constant (*K*_D_ = 0.41 nM) of the interaction

A nitrocellulose filter binding assay assessed Apt-4’s specificity. FAM-labeled Apt-4 was incubated with TIMP-1 and other proteins (AFP, CA199, CEA, BSA, 8His peptide), filtered through a nitrocellulose membrane, and fluorescence intensity measured. Figure 2A shows that Apt-4 had high specificity for TIMP-1 with minimal binding to other proteins. Binding kinetics analysis using BLI technology on the Octet R4 system revealed Apt-4’s strong binding affinity for TIMP-1 (0.41 nM) and a slow off-rate (Fig. 2B).

### Aptamer structure and truncation

**Fig. 3:**
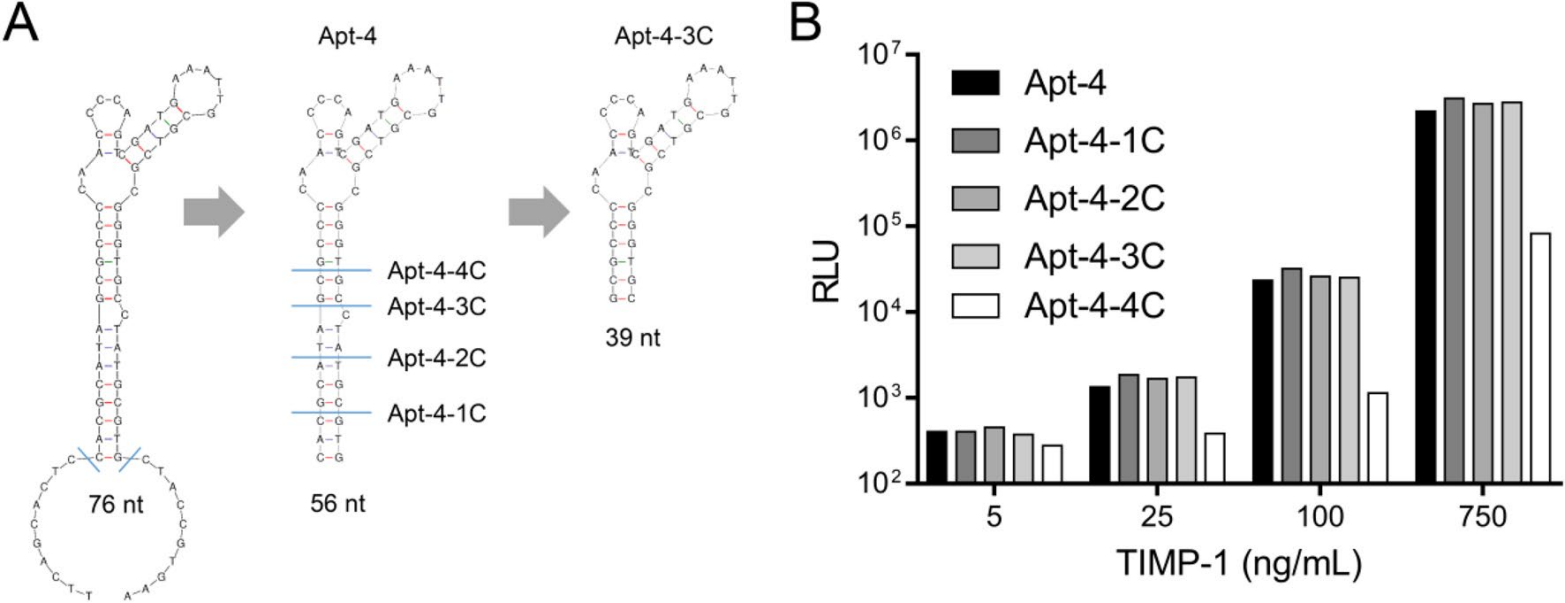
**A** The predicted secondary structure of the aptamer folded by mfold software. The blue lines stand for the truncation site. **B** The binding capability of the truncated aptamers was evaluated using a chemiluminescent immunoassay for TIMP-1 detection

The predicted secondary structure of Apt-4 is shown in Fig. 3A. The full aptamer retained a hairpin structure after removing non-essential primer bases, resulting in a 56-nucleotide aptamer. Truncated variants (Apt-4-1C, Apt-4-2C, Apt-4-3C, and Apt-4-4C) with differing stem lengths were evaluated via a chemiluminescent immunoassay. Figure 3B indicates that only Apt-4-4C exhibited decreased signal intensity, confirming that a six-base pair stem is crucial for binding.

### Chemiluminescent aptasensor immunoassay

**Fig. 4:**
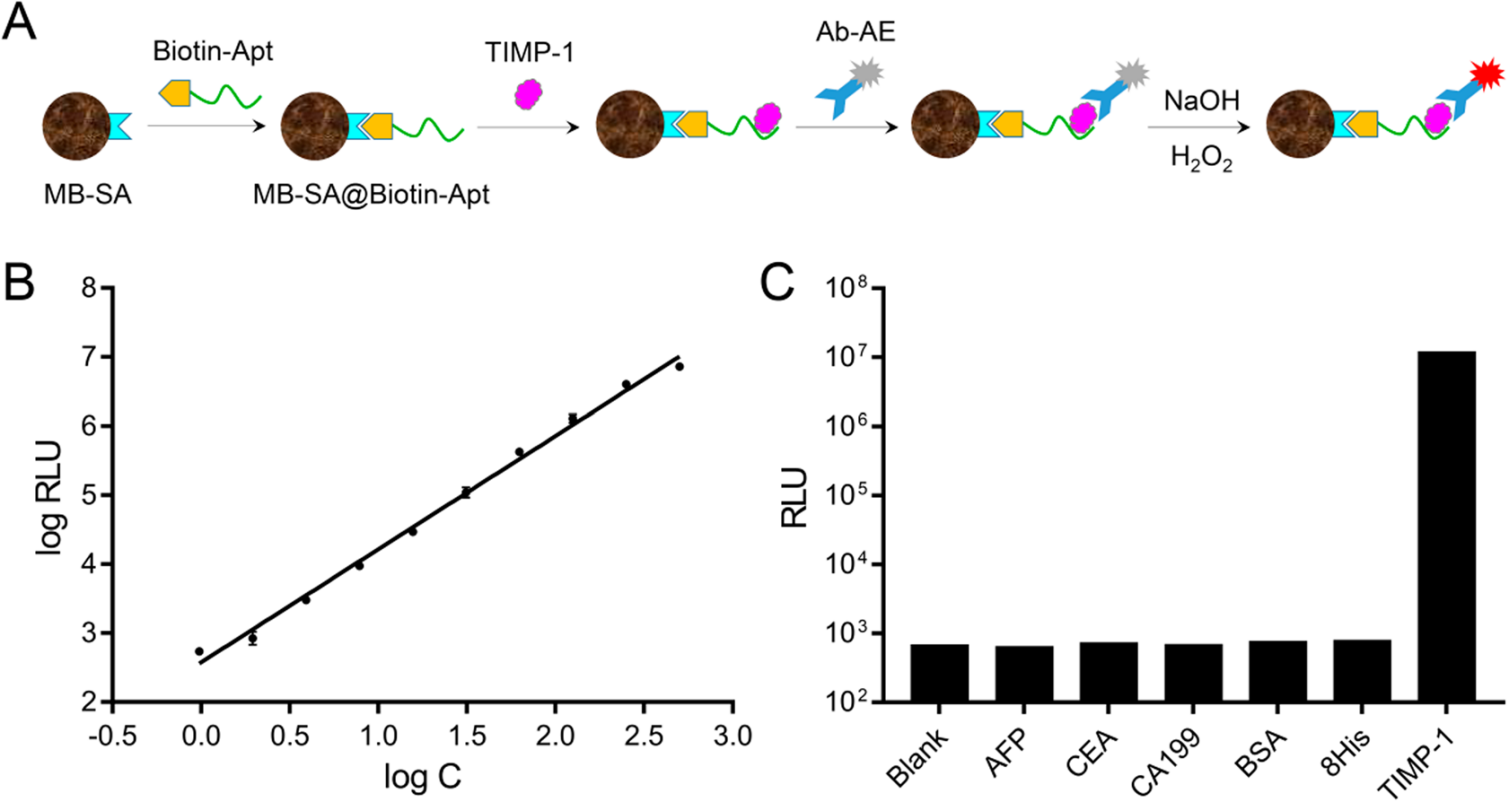
**A** Schematic diagram of the chemiluminescent aptasensor immunoassay. **B** Standard curves for aptasensor. **C** The RLU responses of the aptasensor towards different molecules

A chemiluminescent aptasensor was designed using biotin-modified Apt-4 immobilized on streptavidin magnetic beads (MB-SA@Biotin-Apt) to capture TIMP-1, followed by detection with acridinium ester-labeled antibody (Ab-AE). Chemiluminescence was measured by incubating with NaOH and H_2_O_2_, showing a linear relationship between relative light units (RLU) and TIMP-1 concentrations (1–500 ng/mL) (Fig. 4A, 4B). Specificity tests demonstrated negligible interference from other molecules (AFP, CEA, CA199, BSA, 8His peptide) (Fig. 4C).

### Clinical sample analysis

**Fig. 5:**
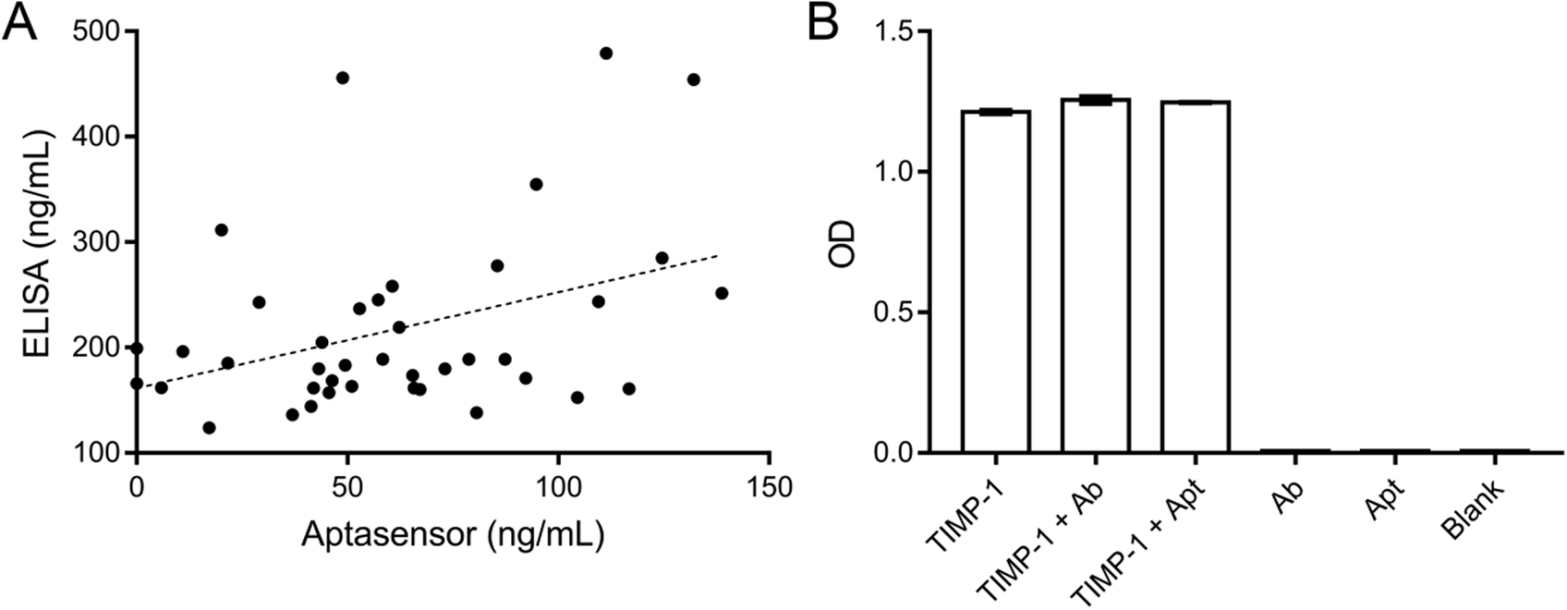
**A** Correlations between the chemiluminescent aptasensor and ELISA. TIMP-1 levels of 40 sera samples were assayed by the chemiluminescent aptasensor and ELISA. Linear correlation was observed in the (X, Y) scatter plot (R^2^ = 0.1446). **B** Inhibition of ELISA test to TIMP-1 by the antibody (Ab) and aptamer (Apt)

The chemiluminescent aptasensor quantified TIMP-1 in 40 human sera samples, compared with enzyme-linked immunosorbent assay (ELISA) results. Figure 5A shows a poor correlation (R^2^ = 0.1446) between the two methods. A competitive inhibition experiment shows that the addition of the antibody or aptamer has no significant inhibition of the ELISA test, suggesting that the aptamer and antibody recognize different TIMP-1 epitopes, leading to detection of different molecular forms (Fig. 5B).

## Discussion

The SELEX process employed in this study effectively enriched high-affinity aptamers for TIMP-1, underscoring the importance of optimizing selection conditions to enhance target-specific characteristics. The use of high ionic strength (500 mM NaCl) and 50% serum in the selection buffer mitigated non-specific electrostatic interactions and improved the aptamers’ specificity, stability, and adaptability. These adjustments were crucial, as demonstrated by the qPCR amplification curves and high-throughput sequencing results, which showed progressive enrichment and decreased diversity of the ssDNA library. The qPCR amplification curve was used for monitoring the library diversity during the progress of aptamer selection [17]. In the initial selection rounds, the ssDNA library consisted of plenty of random oligonucleotides. As the amplification reached a plateau, the reaction components (primers or dNTP) were consumed and the amplification was limited. The plenty of diverse DNA sequences mismatched in the subsequent qPCR cycles resulted in the drop of fluorescence (Fig. 1A, round 1–3). Along with the selection round, the ssDNA library was enriched and the library diversity decreased. The mismatch of the DNA sequences in the qPCR plateau phase reduced, resulting in the decrease (Fig. 1A, round 4–6) and disappearance (Fig. 1A, round 7) of the fluorescence drop. The high-throughput sequencing results of the ssDNA library also confirmed the results. As shown in Fig. 1B, the top 100 sequences were enriched from 11% (round 3) to 61% (round 7). The top 10, 100, and 1000 sequences all display the abundance enhancement during the selection round. This approach aligns with the methodologies employed by other researchers, such as subsequent adaptations in SELEX protocols aimed at reducing non-specific binding and improving aptamer functionality [18].

Apt-4 exhibited exceptional specificity for TIMP-1, with minimal cross-reactivity with other proteins, indicating high selectivity. This high specificity is a critical attribute, as reported by other studies on aptamers targeting specific proteins, where achieving high specificity has been a significant challenge [22, 23]. Binding kinetics analysis via BLI technology further confirmed Apt-4’s strong affinity (0.41 nM) and slow off-rate for TIMP-1, essential for stable and prolonged binding in clinical applications. These results are consistent with findings from research on aptamers targeting cancer biomarkers, where high affinity and low dissociation rates are crucial for diagnostic applications [24, 25].

The structural analysis of Apt-4 identified critical elements for aptamer-target binding. The stem-loop configuration was found to be vital, with a six-base pair stem necessary for maintaining binding efficiency. Truncated variants demonstrated that reducing the stem length beyond this threshold significantly decreased binding capability. This insight into structural requirements is in line with the findings of studies on the structural optimization of aptamers, such as those targeting thrombin and protein tyrosine kinases, where specific structural features are essential for high-affinity binding.

The chemiluminescent aptasensor developed in this study demonstrated high sensitivity, a broad dynamic range, and rapid detection capabilities, making it suitable for clinical diagnostics. The linear response to TIMP-1 concentrations and high specificity against other proteins underscore its potential utility. Similar advancements in aptasensor technology have been reported by other researchers, such as the development of aptamer-based sensors for the detection of various biomarkers, including those for infectious diseases and cancer, where high sensitivity and specificity are paramount.

However, the poor correlation between the aptasensor and ELISA results poses a significant challenge in clinical assay development (R^2^ = 0.1446). This discrepancy is not uncommon; similar results have been reported in the literature for other targets such as CD97, mesothelin, HSP70, and ANGPTL3 [26]. The competitive inhibition experiment suggested that the aptamer and antibody used in the aptasensor and ELISA might recognize different epitopes or molecular forms of TIMP-1, leading to variations in detected levels. TIMP-1 exists in various molecular forms in the body, including uncomplexed TIMP-1 and complexes with other proteins such as pro-MMP-9, CD63, LRP1, and β1 integrin [1]. This highlights a critical area for future research, emphasizing the need for a deeper understanding of the molecular diversity of TIMP-1 and the epitope specificity of the detection methods.

Future research should focus on these variations and refine the aptamer selection process to enhance epitope specificity. Additionally, integrating multi-epitope recognition in aptasensors could improve correlation with conventional antibody-based assays and expand the clinical applicability of aptamer-based diagnostics. This approach is supported by recent studies that have explored multi-target aptamer strategies to improve diagnostic accuracy and reliability. Efforts should also be made to develop standardized protocols and validation procedures to ensure consistency and reliability in clinical settings, aligning with the broader goals of translational research in biomarker detection and clinical diagnostics.

## Conclusions

In conclusion, the ssDNA aptamer targeting for TIMP-1 has been successfully developed, showing high specificity and affinity (*K*_D_ = 0.41 nM) and a very slow off rate. Based on the selected aptamer, a chemiluminescent aptasensor for TIMP-1 detection was constructed. The aptasensor shows high specificity for TIMP-1 and can detect TIMP-1 in human serum, which has great potential for clinical applications. Unfortunately, quantification in 40 sera samples with the aptasensor showed poor correlation with conventional ELISA, which may due to the complex forms of TIMP-1 and the different bidnding site recognised by the aptasensor and ELISA. During TIMP-1 detection, the complex forms of TIMP-1 and the binding site should be considered, and the detection strategy should be carefully desigened. We expect that this work will contribute to the development of aptamer’s application in clinical detection and diagnosis.

## Data Availability

The datasets used and/or analyzed during the present study are available from the corresponding author on reasonable request.

## Abbreviations

TIMP-1: Tissue inhibitor of metalloproteinases 1
MMPs: Matrix metalloproteinases
SELEX: Systematic evolution of ligands by exponential enrichment
ssDNA: Single stranded DNA
EDC: 1-Ethyl-3-(3-dimethylaminopropyl) carbodiimide hydrochloride
NHS: N-hydroxysuccinimide
DPBS: Dulbecco’s phosphate buffered saline solution
ePCR: Emulsion PCR
qPCR: Quantitative PCR
RLU: Relative light units
ELISA: Enzyme-linked immunosorbent assay
BLI: Biolayer interferometry
